# Psychological morbidity and substance use among patients with hypertension: a hospital-based cross-sectional survey from South West Ethiopia

**DOI:** 10.1186/s13033-016-0108-0

**Published:** 2017-01-03

**Authors:** Matiwos Soboka, Esayas Kebede Gudina, Markos Tesfaye

**Affiliations:** 1 Department of Psychiatry, College of Health Sciences, Jimma University, Jimma, Ethiopia; 2 Department of Internal Medicine, College of Health Sciences, Jimma University, Jimma, Ethiopia

**Keywords:** ‘Psychological morbidity’, ‘Substance use’, Hypertension, Ethiopia, ‘Mental health service’

## Abstract

**Background:**

Psychological morbidity and substance use disorders have been linked to cardiovascular diseases; affecting patients’ medical outcome and quality of life. However, little is known about psychological morbidity and substance use among patients with hypertension in Ethiopia. Therefore, we aimed to assess psychological comorbidity and substance use among hypertensive patients in Southwest Ethiopia.

**Methods:**

A cross-sectional study was conducted among 396 hypertensive patients on follow-up at Jimma University Teaching Hospital in Ethiopia during the study period. Structured questionnaires were used to assess alcohol use, khat chewing and cigarette smoking. Psychological morbidity was assessed using the Kessler-6 scale. Multiple logistic regression analysis was carried out to identify the independent association between outcome and explanatory variables.

**Results:**

The prevalence of psychological morbidity among hypertensive patients was 31.6%. Of the total participants, 31 (7.8%) of them had alcohol use disorders and 79 (19.9%) of them were using khat regularly at the time of the study. Singles were more likely to have psychological morbidity than married participants (AOR = 4.72; 95% CI 1.83, 12.20, p = 0.001), whereas those who were able to ‘read and write’ were less likely to have psychological morbidity than non-literate ones (AOR = 0.46; 95% CI 0.24, 0.89, p = 0.02). However, no association was seen between psychological morbidity and substance use (khat chewing, alcohol use and cigarette smoking), belief about hypertension, ever discontinuation of medication and lifestyle (exercise, salt consumption).

**Conclusion:**

Psychological morbidity and substance use are prevalent among hypertensive patients on follow-up at the hospital. The findings of the study imply that there is a need for further studies to understand the effect of psychological morbidity on the clinical outcomes of hypertensive patients.

## Background

Currently, the global burden of disease is shifting from communicable disease to non-communicable diseases. The rising burden from mental and behavioural disorders is increasing from time to time [1]. Mental and behavioural disorders are common, affecting more than 25% of all people at some time during their lives [2]. A significant proportion of the world’s health problems in both high-income and low to middle-income countries arises from mental, neurological and substance use disorders [1]. Mental and substance use disorders were the leading global cause of all non-fatal burden of disease [3, 4]. Mental, neurological and substance use disorders also accounted for 10.4% of global disability-adjusted life year (DALYs), 2.3% of global YLLs and 28.5% of global years lived with disability (YLDs) [5].

Global burden of mental and substance use disorders were greater than the burden of HIV/AIDS, tuberculosis, diabetes and traffic accident [3, 6]. The burden associated with common mental disorders mostly affect adolescent and early to middle age groups [3].

Similarly, the burdens associated with cardiovascular diseases are common all over the world and affecting productive age groups. In 2010, hypertension was the leading risk factor for disability adjusted life years (DALYs) [7, 8]. Cardiovascular disease and mental health conditions are the dominant contributors to the global burden of non-communicable diseases (NCDs) [7]. World Health Organization (WHO) estimates that 25% of all patients using a health service suffer from at least one mental, neurological or behavioural disorder most of which are undiagnosed or untreated [9]. Patients with chronic medical illness like hypertension may develop psychological problems resulting from difficulty in adjusting their aspirations, lifestyle and employment [10]. There is strong association between psychological morbidity and substance use like alcohol, khat and cigarette smoking [11–15]. Psychological morbidity and substance use may reduce patients’ adherence to antihypertensive medications [9, 16–18]. Furthermore, psychological morbidity is reported to have negative effects on quality of life [19] and sleep pattern [20] among patients with hypertension. Similarly, there is strong association between psychological morbidity and hypertension [19].

In Ethiopia, even though several studies have been carried out concerning psychological morbidity and substance use among different communities; little is known about the psychological comorbidity and substance use among hypertensive patients. Therefore, assessing psychological morbidity and substance use among patients with hypertension is crucial in informing policy and programs so that comprehensive care for patients with hypertension can be realized. This study aimed to assess psychological morbidity and substance use among patients with hypertension.

## Methods

### Study area

A cross-sectional study was conducted at Jimma University Teaching Hospital (JUTH) hypertension clinic. JUTH is a referral hospital for over 15 million people located in Southwest Ethiopia. Hypertension clinic is one of the many chronic follow-up clinics of the hospital. At the time of this study, over 2000 patients were on follow-up care for hypertension. The study was conducted over a 6 month period from April 2014 to September 2014.

### Instruments

#### Outcome variable: psychological morbidity

The Kessler 6 scale (K-6), which has been translated into Amharic and validated in Ethiopia [22], was used to measure psychological morbidity (depressive, and anxiety symptoms). The Amharic version of the K-6 has been demonstrated to have a sensitivity and specificity of 84.2 and 82.7%, respectively, at a cut-off point of 5 or greater to screen for symptoms of psychological morbidity [22]. Kessler 6 was translated into Afan Oromo for those participants who were unable to speak Amharic.

#### Explanatory variables

##### Socio-demographic characteristics

A structured questionnaire was used to assess socio-demographic characteristics of participants (age, gender, marital status, educational status, occupation, religion, place of residence), patients’ knowledge about hypertension, and self-care practices (exercise, salt use).

##### Clinical characteristics

Some of clinical characteristics like years lived with hypertension and hypertension related morbidity were collected from medical records of the patients. Participants were also interviewed to assess for past history of mental illness, history of hypertension, its treatment and adherence.

##### Substance use disorders

*Alcohol use disorders (AUD)* was assessed using the four item CAGE questionnaire (cut down, annoyed, guilty, eye opener) [23]. CAGE is short and easily applied in clinical practice with reported sensitivity and specificity at cut-off score ≥2 of 0.71 and 0.90 respectively. A participant who scored two or more on the CAGE was classified as having AUDs [23].

*Cigarette smoking* a self-report questionnaire was used to assess cigarette smoking (current smoker/non-smoker and the number of cigarettes smoked). Those participants who smoked at least one cigarette per day were considered to be cigarette smokers.

*Khat use* a structured questionnaire was used to assess the pattern of khat chewing including frequency. In this study, current khat use was defined as chewing khat during the month prior to the interview.

### Sampling procedure

All eligible adult attendees of the hypertension clinic at JUTH during the study period were invited consecutively to participate in the study. However, patients with severe medical or mental illness that make the interview difficult, and those who were younger than 18 years were excluded.

### Data collection procedures

Data collection was carried out after the questionnaires had been pretested on a sample (5% of the total sample) of patients with hypertension attending the hypertension clinic at JUTH. Those patients who had participated in the pre-test were not included in the main study. Data were collected by four registered nurses and one health officer after 1 day of training on administration of the study instruments. Data collection was supervised by the principal investigators. The supervisors monitored data quality and checked all questionnaires for completeness.

### Data analysis

After double data entry, data were exported from Epi-Data (version 3.1) and analysed using the Statistical Package for Social Sciences (SPSS, version 20). The outcome and explanatory variables were entered into a bivariate logistic regression analysis, one by one, in order to estimate the strength of association using odds ratios (OR). All variables associated with psychological morbidity and with a p value of less than 0.25 were entered together into a multivariable logistic regression in order to control for potential confounders. Age was analysed as a continuous variable. Variables with p value <0.05 in the multivariate analyses were considered significant predictors of psychological morbidity.

### Ethical considerations

Ethical clearance was obtained from the Ethical Review Board of Jimma University. Written informed consent was obtained from each of the study participants prior to data collection. The anonymity of the study participants was kept at all stage of data processing and analysis.

## Result

### Participants’ characteristics

A total of 401 patients with hypertension were approached to participate in this study and 396 of them agreed to participate with response rate of 98.8%. The mean age of participants was 54 ± 12.3 years and ranged from 23 to 87 years. Of the patients participated in the study, 57.3% (n = 227) of them were female. The majority of the study participants were married (77%, n = 305) and urban dwellers (70.2%, n = 278). More than one-third (37.9%, n = 150) of the study participants were non-literate. Approximately, half of the study participants were Coptic Christians (48.5%, n = 192) followed by Muslims (38.1%, n = 151) (see Table 1).

**Table 1:** Socio-demographic characteristics of patients with hypertension on follow up at JUTH during April 2014 to September 2015

Variables	Frequency	%
Gender		
Male	169	42.7
Female	227	57.3
Marital status		
Single	23	5.8
Married	305	77
Other	68	17.2
Educational status		
Non-literate	150	37.9
Read and write	91	23
Primary	67	16.9
Secondary	59	14.9
Tertiary	29	7.3
Occupation		
Farmer	91	23
Teacher	19	4.8
Merchant	51	12.9
House wife	93	23.5
Daily laborer	107	27
Others	35	8.8
Religion		
Muslim	151	38.1
Orthodox	192	48.5
Protestant	41	10.4
Others	12	3
Place of residence		
Rural	118	29.8
Urban	278	70.2

### Prevalence of psychological morbidity and substance use disorders

Out of the total participants, 31.6% (n = 125) of them had psychological morbidity and 5.1% (n = 20) had past history of mental illness. Out of patients with psychological morbidity; 6.4% (n = 8), 1.6% (2), 21.6% (n = 27) of them were identified to use alcohol, tobacco and khat respectively. Nearly one-fifth (19.9%, n = 79) of the study participants were current khat users. Of khat users, 45.6% (n = 36) of them were chewing khat 2–3 times a week, and 35.4% (n = 28) of them were chewing it daily. Alcohol drinking was reported by 7.8% (n = 31) of the participants, and all of them were found to have alcohol use disorders. Only few percent (1.8%, n = 7) of the total participants reported that they were current smokers.

### Factors associated with psychological morbidity

Single participants had nearly five times increased odds of having psychological morbidity compared to married participants (COR = 4.78, 95% CI 1.95, 11.67). Participants with read and write educational status had 54% times less likely to have psychological morbidity compared to participants who were illiterate (COR = 0.46, 95% CI 0.25, 0.84). The odds of having psychological morbidity among patients who had history of antihypertensive medication non-adherence was 1.72 time higher than that of patients who had no history of medication non-adherence (COR = 1.72, 95% CI 1.00, 2.95) (see Table 2).

**Table 2:** Factors associated with psychological morbidity among patients with hypertension on follow up at JUTH during April 2014 to September 2015

Variables	Psychological morbidity		p value	OR	95% CI	
	Yes N (%)	No N (%)			Lower	Upper
Gender						
Male	50 (29.6)	119 (70.4)		Reference		
Female	75 (33.0)	152 (67.0)	0.47	1.17	0.76	1.81
Age			0.47	1.17	0.76	1.81
Marital status						
Single	15 (65.2)	8 (34.8)	0.001	4.78	1.95	11.67
Married	86 (28.2)	219 (71.8)		Reference		
Divorced/widowed	24 (35.3)	44 (64.7)	0.25	1.39	0.80	2.42
Educational status						
Illiterate	55 (36.7)	95 (63.3)		Reference		
Read and write	19 (20.9)	72 (79.1)	0.01	0.46	0.25	0.84
Primary school	23 (34.3)	44 (65.7)	0.74	0.93	0.49	1.65
Secondary/tertiary school	28 (31.8)	60 (68.2)	0.45	0.81	0.46	1.41
Occupation						
Farmer	29 (31.9)	62 (68.1)	0.88	1.05	0.57	1.92
Teacher	5 (26.3)	14 (73.7)	0.69	0.80	0.27	2.41
Merchant	21 (41.2)	30 (58.8)	0.20	1.57	0.79	3.14
Housewife	28 (30.1)	65 (69.9)	0.91	0.97	0.53	1.77
Daily laborer	33 (30.8)	74 (69.2)		Reference		
Others	9 (25.7)	26 (74.3)	0.57	0.78	0.33	1.84
Religion						
Orthodox	51 (33.8)	100 (66.2)		Reference		
Muslim	58 (30.2)	134 (69.8)	0.48	0.85	0.54	1.34
Protestant	12 (29.3)	29 (70.7)	0.59	0.81	0.38	1.72
Others	4 (33.3)	8 (66.7)	0.98	0.98	0.28	3.41
Place of residence						
Rural	41 (34.7)	77 (65.3)	0.38	1.23	0.78	1.94
Urban	84 (30.2)	194 (69.8)		Reference		
Hypertension is curable disease						
Yes	101 (31.9)	216 (68.1)		Reference		
No	24 (30.4)	55 (69.6)	0.80	0.93	0.55	1.59
Hypertension is deadly disease						
Yes	110 (31)	245 (69.0)	0.47	0.78	0.40	1.53
No	15 (36.6)	26 (63.4)		Reference		
Hypertension medication is habit forming						
Yes	78 (31.2)	172 (68.8)	0.84	1.05	0.68	1.62
No	47 (32.2)	99 (67.8)		Reference		
Ever discontinuation of medication						
Yes	28 (41.8)	39 (58.2)	0.05	1.72	1.00	2.95
No	97 (29.5)	232 (70.5)		Reference		
Regular exercise						
Yes	90 (31.8)	193 (68.2)		Reference		
No	35 (31.0)	78 (69.0)	0.87	0.92	0.60	1.54
Salt consumption						
Yes	79 (30.4)	181 (69.6)	0.87	1.04	0.65	1.66
No	46 (33.8)	90 (66.2)		Reference		
History of mental illness						
Yes	9 (45.0)	11 (55.0)	0.49	0.85	0.55	1.33
No	116 (30.9)	260 (69.1)		Reference		
Years lived with hypertension						
<3 years	34 (25.8)	98 (74.2)		Reference		
3 to <5 years	31 (35.6)	56 (64.4)	1.19	1.60	0.89	2.87
5 to <10 years	39 (37.1)	66 (62.9)	0.06	1.70	0.98	2.63
10 years and more	21 (29.2)	51 (70.8)	0.60	1.19	0.63	2.25

After adjusting for potential confounders using multivariate logistic regression, being non-literate and single were associated with psychological morbidity. The odds of having psychological morbidity among singles was more than four times higher compared to married participants (AOR = 4.72, 95% CI 1.83, 12.20). Similarly, participants with educational status of read and write were less likely to have psychological morbidity by 54% compared to non-literate participants (AOR = 0.46, 95% CI 0.24, 0.89). However, psychological morbidity was not associated with gender, age, occupation, years lived with hypertension and ever discontinuing medications (see Table 3).

**Table 3:** Multivariate logistic regression of factors associated with psychological morbidity among patients with hypertension who were on follow up at JUTH during April 2014 to September 2015

Variables	p value	AOR	95% CI	
			Lower	Upper
Gender				
Male		Reference		
Female	0.89	1.04	0.56	1.93
Age	0.91	1.00	0.98	1.02
Marital status				
Single	0.001	4.72	1.83	12.20
Married		Reference		
Divorced/widowed	0.64	1.17	0.62	2.20
Educational status				
Illiterate		Reference		
Read and write	0.02	0.46	0.24	0.89
Primary school	0.70	0.87	0.44	1.74
Secondary/tertiary school	0.60	0.83	0.42	1.65
Occupation				
Farmer	0.68	1.16	0.58	2.30
Teacher	0.93	0.95	0.30	2.99
Merchant	0.16	1.73	0.81	3.70
Housewife	0.82	0.92	0.44	1.90
Daily laborer		Reference		
Others	0.51	0.73	0.28	1.87
Years lived with hypertension				
<3 years		Reference		
3 to <5 years	0.23	1.47	0.78	2.75
5 to <10 years	0.07	1.72	0.96	3.09
10 years and more	0.76	1.14	0.56	2.24
Ever discontinuation of medication				
Yes	0.07	1.70	0.96	3.04
No		Reference		

## Discussion

Psychological morbidity and substance use problems were found to be highly prevalent among hypertensive patients on follow-up at Jimma University hospital. Marital status and educational status were found to be independently associated with the occurrence of psychological morbidity. However, there was no association between substance use and psychological morbidity.

The prevalence of psychological morbidity found in this study (31.6%) was higher than the findings of community based studies done among hypertensive patients in England (15.7%) and former Soviet Union (9.9%) [21, 24]. Similarly, the prevalence of psychological morbidity found in this study was higher than the finding of similar study done in West Africa (10.8%) [25]. The discrepancy between the four studies might be due to the difference in the tools used to assess psychological morbidity (Kessler-6 vs GHQ-12). Moreover, the study done in West Africa excluded patients with past history of mental illness and complication from hypertension.

In this study, psychological morbidity was associated with being less educated (read and write) which was similar with the study finding done in former Soviet Union [21]. Also, high prevalence of psychological morbidity among illiterates (43.8%) was reported previously among Jimma town community [13]. Persons with less education might experience other socioeconomic disadvantages which contribute to poor mental health. The lack of association between lower psychological morbidity and an educational level of primary school or above could be due to underpowered sample size in these groups. Similarly, being single was associated with psychological morbidity which was in agreement with a community based study done in Jimma town [13]. Also, the odds of having psychological morbidity among single participants was more than four times higher compared to married participants. This could be due to the fact that being married is one of the protective factors for mental illness [26] because social support from spouse could reduce the effects of psychosocial stressors and protect individuals from psychological morbidity [28].

The prevalence of current khat chewing (19.9%) found in our study was lower than the finding of community based study done in Jimma town (37.8%) [13]. The discrepancy may be due to difference between study participants. The patients in our study who were interviewed in the hospital setting might tend to under-report use of khat unlike community participants who were interviewed in their homes. Also, the patients may have received information from health professionals not to chew khat. In our study, even though the prevalence of khat chewing among patients with psychological morbidity was high, there was no association between psychological morbidity and khat chewing which might be due to underreporting of khat use. Hypertensive patients with both psychological morbidity and substance use who are likely be non-compliant [27] or dropout from follow-up introducing selection bias.

The prevalence of alcohol use and use disorder found in this study (7.8%) was lower than the findings from community based study done in Jimma town (62.4%), Gurage Zone (21%) and Buta jira (16%) Ethiopia [29–31]. Our sample is composed of older adults who have been on follow-up for a medical condition. Substance use is more common among younger population [26, 32]. The study participants had regular contact with health care providers who would have advised them to avoid use of substances. Other factors may include socio-cultural differences and tools used to assess alcohol use and use disorder (unstandardized tool vs CAGE and FAST).

The prevalence of tobacco use found in this study (1.8%) was lower than the community based study done in Jimma town (26.2%) and Eastern Ethiopia (28%), [29, 33]. Also the prevalence of tobacco use found in this study was lower than the overall finding of national tobacco use survey in Ethiopia (4.1%) [34]. The lower prevalence of tobacco use in our study might be due to health professionals’ advise not to smoke tobacco compared to the national wide review of tobacco use. Furthermore, social desirability bias may have contributed to underreporting of tobacco use in our sample.

Gender, age, occupation, substance use, years lived with hypertension, ever discontinuation of medication, belief about hypertension and lifestyle (exercise, salt consumption) were not associated with psychological morbidity. There is a general observation that women have higher prevalence of depressive and anxiety disorders [26]. It may be that women with hypertension and comorbid mental health conditions may have poorer access to hypertension treatment. However, community based studies are needed to explore this. On the other hand, social desirability bias and underpowered sample size may have contributed to the lack of association.

## Limitations

Social desirability bias may be a limitation of the study as patients may minimize or not disclose about their khat use, alcohol use and smoking. The use of a screening tool for psychological morbidity may have led to over estimation of prevalence. However, the Kessler-6 has been validated in a similar setting and does not include physical symptoms which might have been due to physical illness or medications. Similarly, Kessler-6 was not validated in Ethiopia among hypertensive patients. Also, lack of standard questionnaire for khat chewing was important limitation of the study. In this study standard tool was no used to assess antihypertensive adherence. Our sample being selected from a referral hospital limits the generalizability of the study findings to all hypertensive patients in Southwest Ethiopia. Because of the cross-sectional design, causal associations cannot be established.

## Conclusions

A significant proportion of hypertensive patients were found to have psychological morbidity and substance use problems. These findings may negatively affect treatment outcome of patients with hypertension. The findings of the study imply that there is a need for further studies to understand the effect of psychological morbidity on the clinical outcomes of hypertensive patients.
